# History of the American Medical Association

**Published:** 1855-09

**Authors:** 


### HISTORY OF THE AMERICAN MEDICAL ASSOCIATION.

Dr. N. S. Davis wrote a series of articles for the *New Jersey Medical Reporter*, a year or two since, giving the history of the American Medical Association, in the origin of which it is known he took the deepest interest. These articles have been republished and now lie on our table in the shape of a neat octavo volume, embellished by handsome lithographic likenesses of the distinguished Author and the Presidents of the Association. The history will have interest for the readers of another generation; those of the present day must already be pretty familiar with the origin and labors of this learned body. We do not much fancy the practice of writing biographical sketches while the subjects of them are still alive, as happens to be the case in nearly every instance in the work before us. But we will not scan too critically the merits of a volume meant rather to gratify the friends of the gentlemen whose pictures adorn it than as a substantial contribution to our professional literature.

